# *JAK3* mutations in Italian patients affected by SCID: New molecular aspects of a long‐known gene

**DOI:** 10.1002/mgg3.391

**Published:** 2018-07-21

**Authors:** Gigliola Di Matteo, Maria Chiriaco, Alessia Scarselli, Cristina Cifaldi, Susanna Livadiotti, Silvia Di Cesare, Valentina Ferradini, Alessandro Aiuti, Paolo Rossi, Andrea Finocchi, Caterina Cancrini

**Affiliations:** ^1^ Department of Systems Medicine University of Rome Tor Vergata Rome Italy; ^2^ Department of Pediatrics Children's Hospital Bambino Gesù Rome Italy; ^3^ Department of Biomedicine and Prevention University of Rome Tor Vergata Rome Italy; ^4^ San Raffaele Telethon Institute for Gene Therapy (SR‐TIGET) Pediatric Immunohematology Unit San Raffaele Scientific Institute Milan Italy; ^5^ Vita‐Salute San Raffaele University Milan Italy

**Keywords:** Alu repeats recombination, founder effect, *JAK3* novel mutations, SCID

## Abstract

**Background:**

Mutations in the Janus Kinase 3 (*JAK3*) gene cause an autosomal recessive form of severe combined immunodeficiency (SCID) usually characterized by the absence of both T and NK cells, but preserved numbers of B lymphocytes (T‐B+NK‐SCID). The detection of larger (>100 bp) genomic duplications or deletions can be more difficult to be detected by PCR‐based methods or standard NGS protocols, and a broad range of mutation detection techniques are necessary.

**Methods:**

We report four unrelated Italian patients (two females and two males) with SCID phenotype. Protein expression, functional studies, molecular analysis by standard methods and NGS, and transcripts studies were performed to obtain a definitive diagnosis.

**Results:**

Here, we describe four JAK3‐deficient patients from four unrelated families. The first patient is homozygous for the known c.1951 C>T mutation causing the amino acidic change p.R651W. The other two patients, originating from the same small Italian town, resulted compound heterozygotes for the same g.15410_16542del deletion and two different novel mutations, g.13319_13321delTTC and c.933T>G (p.F292V), respectively. The fourth patient was compound heterozygous for the novel mutations p.V599G and p.W709R. Defective STAT5 phosphorylation after IL2 or IL15 stimulation corroborated the mutation pathogenicity. Concerning g.15410_16542del mutation, probably due to an unequal homologous recombination between Alu elements of *JAK3* gene, microsatellites analysis revealed that both unrelated Pt2 and Pt3 and their carrier family members shared the same haplotype. These data support the hypothesis of a founder effect for the g.15410_16542del mutation that might have inherited in both unrelated families from the same ancient progenitor.

**Conclusion:**

Different molecular techniques are still required to obtain a definitive diagnosis of AR‐SCID particularly in all cases in which a monoallelic mutation is found by standard mutation scanning methods.

## 1 INTRODUCTION

Severe combined immunodeficiency (SCID) is a heterogeneous group of primary immunodeficiency disorders characterized by defect of both T and B lymphocyte development and function (Buckley, 2000; O'Shea et al., 2004). SCID patients, during the first months of life, are characterized by recurrent severe and opportunistic infections, intractable diarrhea, and failure to thrive resulting in a fatal outcome without hematopoietic stem cell transplant (HSCT) or gene therapy (GT) treatment (Alsum et al., 2012; Tezcan et al., 2005; Yu et al., 2011). The X‐linked SCID (X‐SCID), caused by mutations in *IL2RG* gene encoding the common gamma‐chain (γ‐chain), is the most frequent form of the disease and its clinical phenotype is comparable to that of autosomal recessive SCID (AR‐SCID) caused by Janus kinase 3 (*JAK3*) gene mutations (OMIM: * 600173). JAK3 has a key role in the signaling downstream of the common gamma‐chain subunit that is an essential component of the IL‐2, IL‐4, IL‐7, IL‐9, IL‐15, and IL‐21 receptor complexes; indeed, *JAK3*‐deficiency is the main cause of T‐B+NK‐ AR‐SCID (OMIM: #600802; Wu & Sun, 2011; Rochman, Spolski, & Leonard, 2009).

To date, the majority of JAK3‐SCID patients were compound heterozygous and the remaining patients were homozygous as result of parental consanguinity (Lee et al., 2011; Mjaanes, Hendershot, Quinones, & Gelfand, 2007; Notarangelo et al., 2001; Qamar et al., 2017; Roberts et al., 2004; Scarselli et al., 2016; Stepensky et al., 2016; Uchiyama et al., 2005). Although the most frequent defect resulted in a marked reduction or complete absence of the JAK3 protein expression/function, patients with missense mutations and a reduced JAK3 functional activity could show an atypical and less severe immunological phenotype (Frucht et al., 2001; Scarselli et al., 2016).

In this study, we describe four Italian patients in which we found five new mutations in *JAK3* gene. For one of these, the g.15410_16542del, we suggest a mechanism of recombination between Alu elements as cause of deletion. This mutation was found in two unrelated patients and a possible founder effect was investigated. Protein and molecular studies were performed to have a definitive diagnosis in these patients.

## 2 MATERIALS AND METHODS

### 2.1 Patients

We report four unrelated Italian patients (2 females and 2 males) with SCID phenotype. In Table 1, we reported immunological data from all patients. The pedigree analysis of the four different families did not reveal any case of parental consanguinity. Informed consent was obtained from the patients’ and controls’ parents for immunological and genetic investigation.

**Table 1:** Immunological characteristics of severe combined immunodeficiency (SCID) patients

Test	Patients				
	Pt1^a^	Pt2	Pt3	Pt4	Normal value^b^
Lymphocyte subsets (cells/μl)					
ALC	351	2,420	5,490	4,990	5,690 (3,320–7,006)
CD3+	73.7	24.2	439.2	733.5	3,833 (2,284–4,776)
CD3+CD4+	42.1	N/D	109.8	40	2,492 (1,523–3,472)
CD3+CD8+	28.0	N/D	274.5	349.3	976 (524–1,583)
CD19+	203.0	2,357	4,940.1	4,126.7	1,123 (776–2,238)
CD16+/CD56+	28.0	0.0	109.8	70.0	381 (230–801)
Serum immunoglobulins (mg/dl)					
IgG	7	31	257	91	633–1,016
IgA	7	3	30	<5	41–315
IgM	5	28	22	15	56–261
Lymphoproliferation test (10^3^ cpm)					
PHA (2.5 μg/mL)	0.8	1.6	2.1	N/D	>30 cpm
OKT3 (1.5 μg/mL)	0.7	2.7	0.4	N/D	>20 cpm
PWM (0.35 μg/mL)	4.3	3	1.5	N/D	>18 cpm

ALC, absolute lymphocyte count at diagnosis; N/D, not done.

a 59% of PBMCs were derived from maternal T cells.

b Age matched (3‐12 months) absolute count of lymphocytes subsets (n×10^6^/L= cells/μl): median and (10th to 90th) percentiles (Tosato et al., 2015).

Patient 1 (Pt1) was a boy born preterm at 33 weeks of gestational age. He was mechanical ventilated because of neonatal respiratory distress. He received both parenteral nutrition and breastfeeding. He was finally discharged from the hospital in good general conditions. Immunological or other clinical or instrumental data concerning the period after this first discharge at the age of 6 weeks were not available. The CBC at the birth was as follows: GB 3700, Hb 17,4, PLT 266000, GR 4.680.000 and at discharge: GB 4900, Hb 8.1, PLT 456.000, GR 2580.000 and none earlier diagnosis had been made.

The presence of about 59% of peripheral blood mononuclear cells (PBMCs) derived from transplacentally acquired maternal T cells and a partial expression of the protein could lead to a less severe phenotype in this patient (Tezcan et al., 2005). At the age of 8 months, he presented impetigo treated with local and oral antibiotic, recurrent diarrhea and otitis due to *proteus mirabilis* poor responsive to antibiotics therapy. During this hospitalization, the total lymphocyte count of 315/mm^3^ (CD3 21%; CD4 12%; CD8 8%; CD19 58%; CD57 2%; CD16 8%) and a low dosage of immunoglobulines induced the suspect of an immunodeficit. He was transferred at age of 10 months to our hospital in poor general conditions, to be investigated for immunodeficiency. A chest radiograph and a total body TC were performed in which none particular alteration was highlighted except signs of ongoing otitis and normal mediastinal structures were reported.

Allogeneic haploidentical HSCT from his mother was successfully performed at the age of 12 months. Eleven years after HSCT, the patient is in general good condition and shows adequate immunological function.

Patient 2 (Pt2) was a girl with a history of a phlegmon on the thigh at 4 months of age. She was admitted to our hospital because of severe SRV bronchiolitis with acute respiratory distress, and an immunodeficiency was suspected although a lymphocyte absolute count of 2,420/ml was detected. She underwent a successful HSCT from her HLA‐identical sister at the age of 7 months. Eleven years after HSCT, she maintains good clinical condition and immunoreconstitution.

Patient 3 (Pt3) was a girl hospitalized at 4 months of age due to failure to thrive. She was discharged 3 days later with a suspect of cow milk allergy. Because of persistent vomiting, she was subsequently admitted to our hospital and discharged with a diagnosis of urinary infection. However, she was readmitted a few days later for wheezing and cough. A rapidly progressive respiratory failure with a picture of interstitial pulmonary picture occurred, necessitating mechanical ventilation. The rapid clinical deterioration suggested a diagnosis of SCID confirmed by immunological phenotype and molecular analysis. Bronchoalveolar lavage (BAL) and tracheal aspirate (TA) for bacteria, EBV, CMV, adenovirus, influenzae, parainfluenzae resulted negative. Treatment with antibiotics, intravenous immunoglobulins, trimethoprim‐sulfamethoxazole, fuconazole, acyclovir, and antiviral prophylaxis was promptly started without any improvement. She died one month after haploidentical HSCT.

Patient 4 (Pt4) was a boy born at term. He was well until 4 months of age when he was brought to our attention due to acute respiratory failure, without fever. He had history of long‐lasting cough and a growth arrest in the last month. The chest X‐ray performed revealed an interstitial pneumonia and an antibiotic therapy was started unsuccessfully. Immunological investigations showed a hypogammaglobulinemia and a T and NK lymphopenia despite normal lymphocyte count. Chimerism analysis did not reveal maternal T cells. Due to the detection of Pneumocystis Jirovecii in tracheal aspirate Trimethoprim‐sulfamethoxazole and corticosteroids were started with fast clinical improvement. Allogenic haploidentical HSCT from her mother was successfully performed at the age of 8 months. Seventeen months after HSCT, he did not present severe complications and he showed mix chimerism and normal lymphocytes count.

### 2.2 Molecular studies

*DHPLC and Sequencing:* genomic DNA isolated from peripheral blood of patients and HD using standard protocols (QIAamp DNA Blood kit by QIAGEN GmbH, Hilden, Germany). All 23 exons and exon/intron boundaries of the *JAK3* gene (NG_007273.1; LRG_77) were separately amplified by PCR using specific primers (available on request). PCR reactions were carried out using GoTaq DNA polymerase by standard methods (Promega, Madison, WI). DHPLC was performed on a WAVE analysis system (Transgenomic, Omaha, NE) using different temperature conditions (available on request). Each heteroduplex sample was run on a DnaSept.M column (Transgenomics) and monitored by ultraviolet light (260 nm) using standard protocols (Xiao & Oefner, 2001). Samples with an altered chromatographic profile were sequenced. Direct sequencing was performed using the BigDye Terminator v3.1 Cycle Sequencing Kit (Applied Biosystems, Foster City, CA) and analyzed on an ABI PRISM 3130 and 310 automated sequencers (Applied Biosystems).

*RT‐PCR*: Total RNA was isolated from EBV‐B lymphocytes of both JAK3‐SCID patients and their parents by using Trizol procedure (Invitrogen ‐ Life Technologies, Milano, Italy). Reverse transcription was performed using a single reaction kit (Superscript, Invitrogen ‐ Life Technologies) according to the manufacturer's instructions. Nine overlapping primer pairs (available on request) were used for amplification of the cDNA (NM_000215.3).

### 2.3 Immunoblot analysis

Protein lysates were obtained from EBV‐B lymphocytes from patients and HD using JS1X lysis buffer (50 mM Tris/HCl ph 8, 150 mM NaCl, 1.5 mM MgCl2, 5 mM EGTA, 1% Triton‐X, 10% glycerol, 1 mM PMSF, aprotinin 1 mg/ml, leupeptin 1 mg/ml, pepstatin 1 mg/ml, 1 mM DTT). Lysates were size‐fractionated by SDS‐PAGE gel and then transferred to nitrocellulose membrane (Protran by Schleicher & Schuell‐Bioscience, Dassel, Germany). Membranes were blocked (5% no‐fat milk for 1 h at room temperature) and then incubated with JAK3‐C21‐rabbit primary antibody (Santa Cruz Biotechnology Inc., CA, USA). The antibody‐specific binding was detected using goat anti‐rabbit IgG conjugated with horseradish peroxidase and visualized by ECL (LiteAblot by Euroclone SpA, Switzerland).

### 2.4 Microsatellite Genotyping

Genomic DNA was extracted from PBMC by standard procedures. Four markers were selected from linkage mapping set panel: (Applied Biosystems), with respect to the telomeric and centromeric limits. PCR was performed in according to the manufacturer's instructions. PCR products were run on an ABI 310 automated sequencer (Applied Biosystems), in the presence of ROX GS‐500 fluorescent size standard. GENESCAN 3.1 and GENOTYPER 2.0 software were used for data collection and allele sizing. Four STRs markers were selected for this study and typed in all family members. D19S221 and D19S226 are localized upstream the gene, while D19S556 and D19S931 are localized downstream the gene. We selected the markers from UCSC genome browser (available at http://genome.ucsc.edu/). PCR reactions (95°C for 12 min, followed by 10 cycles of denaturation at 94°C for 15 s, annealing at 55°C for 15 s and 72°C for 30 s, followed by 20 cycles of denaturation at 89°C for 15 s, annealing at 55°C for 15 s and 72°C for 30 s, and a final extension of 72°C for 5 min) were performed using True Allele PCR Premix.

### 2.5 Phospho‐STAT5 analysis

EBV‐B cells from patients and HD were serum‐starved o.n. and stimulated with IL15 (10 ngr/ml) or IL‐2 (104 IU/ml) (Peprotech, NJ, USA) for 10 minutes at 37°C. Cells were then fixed at room temperature in 10% freshly prepared paraformaldehyde (PFA) for 10 minutes, followed by a rinse in ice‐cold methanol. The washed cells were stained with anti‐phospho‐STAT5 (p‐STAT5) Alexa 488 (Y694, clone 47, BD Biosciences) according to the manufacturer's instructions. Samples were acquired on a FACScantoII flow cytometer (BD Biosciences), and data were analyzed using FlowJo software (TreeStar Inc, Ashland, Ore).

### 2.6 Targeted gene design and panels

Two different custom Ion Torrent panels for a total of 42 genes related to a broad spectrum of PID have been designed: 17 known genes related to SCID‐CID phenotypes and some other miscellaneous PID genes (panel 1: 85.85 kb) and 25 for more rare CID genes (panel 2:101.9 kb) (Table S1a and b). Each gene included in the panels has a 10 bp of exon padding to cover the flanking regions of exon's coding sequences (CDS) including the untranslated regions (UTRs).

### 2.7 Ion Torrent library

Five nanograms of gDNA was amplified by using gene panel Primer Pools, AmpliSeq HiFi mix (Thermo Fisher), and 17 amplification cycles as protocol to obtain the libraries. DNA PCR pools for each sample were combined and subjected to primer digestion with FuPa reagent (Thermo Fisher). Libraries were indexed using the Ion Xpress Barcode Adapter Kit. After purification, we quantified the all amplified libraries with the Qubit^®^ 2.0 Fluorometer.

### 2.8 Enrichment

After dilution of all samples at 100 pM, amplicon libraries were pooled for emulsion PCR (ePCR) on an Ion OneTouch System 2TM using the Ion PGM Template OT2 200 kit, according to the manufacturer's instructions. After ePCR, recovered templated Ion Sphere particles (ISPs) were enriched by using the Ion OneTouch ES system. Quality control of all libraries was performed on Qubit^®^ 2.0 Fluorometer.

### 2.9 Ion Torrent sequencing

Ampliseq DesignSamples were subjected to the standard ion PGM 200 Sequencing v2 protocol using Ion 316 v2 chips (Life Technologies).

### 2.10 Ion Torrent bioinformatics analysis

Mapping and variants calling were performed using the ion Torrent suite software v3.6. Sequencing reads were aligned against the USC hg19 reference genome using the program distributed within the Torrent mapping Alignment Program (TMAP) map4 algorithm (Thermo Fisher; https://github.com/iontorrent/TS). The output of sequence alignment program is a BAM file containing mapped reads. The aligned reads were processed for variant calling by using the Torrent Suite Variant Caller TVC program; variants found in Variant Calling Format (VCF) file were annotated using ANNOVAR. The called variants with minimum coverage of 20X, standard Mapping Quality, and Base Phred Quality were examined on Integrative Genome Viewer (IGV) and BIOMART. After applying filtering criteria, all nonsense, frameshift, and canonical splice site variants were evaluated to determine their potential pathogenicity.

## 3 RESULTS

**Figure 1:**
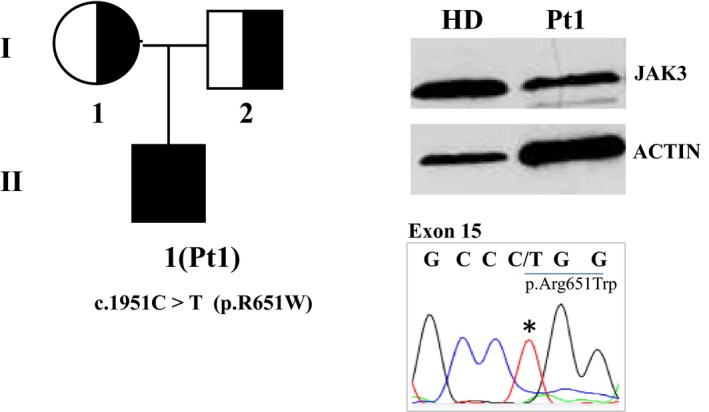
Characterization of JAK3‐Pt1. Genealogical tree, protein expression evaluated by Western blot analysis and the homozygous mutation found by *JAK3* DNA sequencing in Pt1 (NM_000215.3)

In Pt1, JAK3 protein investigation, performed on EBV‐B cells, showed a reduced protein expression compared with HD (Figure 1). Abnormal DHPLC profile (data not shown) was found in exon 15 of *JAK3* gene of patient, and Sanger sequencing revealed the presence of the c.1951 C>T (from the ATG) homozygous substitution that causes the amino acidic change p.R651W (Lee et al., 2011).

**Figure 2:**
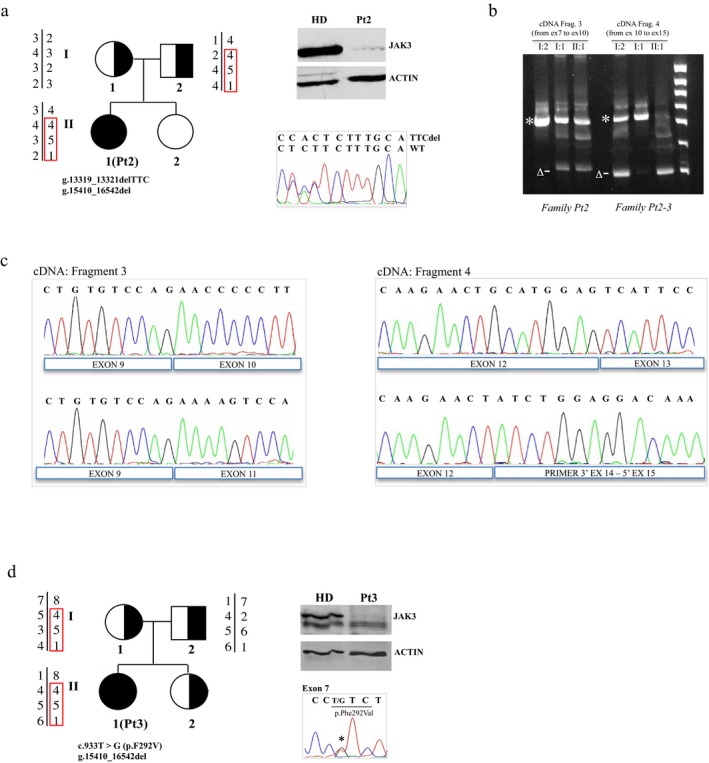
Characterization of JAK3‐Pt2 and JAK3‐Pt3. (a) Genealogical tree of Pt2 with the haplotypes for markers D19S221, D19S226 (localized upstream the gene) and D19S556, D19S931 (localized downstream the gene) are indicated. JAK3 protein expression and the mutation g.13319_13321delTTC (NG_007273.1) confirmed by Sanger sequencing are also indicated. (b) RT‐PCR analysis of JAK3 fragment 3 (FR3: from exon 7 to exon 11) and fragment 4 (FR4: from exon 10 to exon 15) obtained from patient 2 (II:1), her mother (I:1) and her father (I:2). Asterisk and Δ symbols indicate the wild type and the deletion‐derived PCR products, respectively. (c) FR3 and FR4 sequences of Pt2 are showed below and compared with the wild‐type sequence above. The g.13319_13321delTTC deletion caused the skipping of exon 10 in the FR3 and the g.15410_16542del (NG_007273.1) deletion caused the skipping of exons 13 and 14 in FR4. The same pattern was obtained by analyzing the FR4 of Pt3 and her mother (data not shown). (d) Genealogical tree of Pt3 with the haplotypes for markers D19S221, D19S226 and D19S556, D19S931 are indicated together with the JAK3 protein expression and the mutation c.937T>G found by DNA sequencing (NM_000215.3)

**Figure 3:**
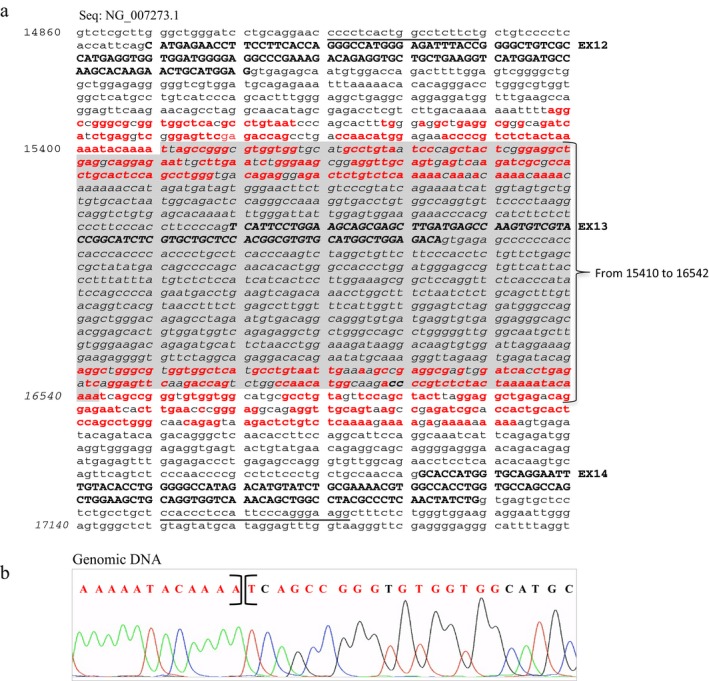
*JAK3* Alu repeat elements. (a) Exons are indicated in bold. The upstream and downstream Alu repeat sequences, delimiting the g.15410_16542del deleted region (in gray), are indicated in red. (b) Sanger sequence of the deleted genomic region amplified by the underlined primers upstream exon 11 and downstream exon 14

Protein analysis performed on EBV‐B cells from Pt2 revealed the absence of the JAK3 protein (Figure 2a). DHPLC profile showed an alteration in exon 10 of the *JAK3* gene of Pt2 and her mother (data not shown), after confirmed by sequencing analysis that revealed the novel g.13319_13321delTTC deletion (NCBI Reference Sequence: NG_007273.1). Further RT‐PCR investigations performed in Pt2 and her parents revealed the skipping of exon 10 caused by the g.13319_13321delTTC deletion inherited from her mother (Fragment 3 in Figure 2b and chromatograms in Figure 2c), and the presence of a second alternative product lacking exons 13 and 14 (Fragment 4 in Figure 2b and chromatograms in Figure 2c) inherited from her father. Only through cDNA studies, we could reveal this deletion otherwise undetectable. A deep genomic investigation revealed the exact deletion endpoints. Two primers mapping upstream of exon 12 and downstream of exon 14 disclosed a novel 1132‐bp deletion (g.15410_16542del), including exon 13 of *JAK3* gene (shown in Figure 3a, b). This deletion was responsible for the skipping of exons 13 and 14 and has been probably generated by two direct Alu repeats, upstream (intron 12), and downstream (intron 13) of the deleted region indicated in Figure 3a.

JAK3 protein was absent also in EBV‐B cells from Pt3 (Figure 2d). The analysis of the genomic DNA by DHPLC performed on patient and her parents showed an atypical profile in the exon 7 in the Pt3 and her father (data not shown). Sequencing revealed the novel missense mutation c.933T>G causing the amino acid change p.F292V, and PolyPhen‐2 web server predicted a possible damaging effect (score of 0.572) for this mutation located in the FERM domain of the protein. In addition, RT‐PCR revealed in Pt3 and her mother the same deletion g.15410_16542del (fragment 4) founded in Pt2 (data not shown). Since Pt2 and Pt3 originated from the same small Italian town, in order to determine whether the mutation g.15410_16542del descended from a single ancestral mutation event or arisen independently, we genotyped their families using four microsatellite markers flanking the *JAK3* gene on chromosome 19 (Figure 2a, d). Interestingly, the same shared haplotype was found in both patients: Pt2 inherited the deletion from her father whereas Pt3 from her mother.

**Figure 4:**
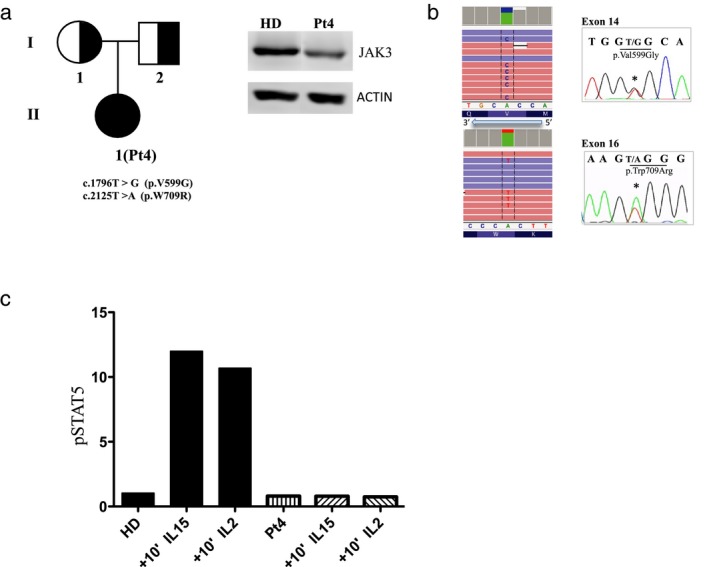
Characterization of JAK3‐Pt4. (a) Genealogical tree and JAK3 protein expression of Pt4. (b) The sequence alignment of the patient's BAM file on IGV (Integrative Genomics Viewer) revealed the indicated mutations confirmed by Sanger. (c) Phospho‐STAT5 (pSTAT5) evaluated in response to IL2 or IL15 stimulation in patients’ EBV‐B cells compared with HD

Protein analysis of Pt4 in Figure 4a revealed a slight decreased expression of JAK3 protein. We identified, by targeted NGS sequencing, two novel mutations: c.1796T>G and c.2125T>A that cause the p.V599G and the p.W709R amino acidic changes, respectively. Both mutations have confirmed by Sanger sequencing in Pt4 and her parents. PolyPhen‐2 web server predicted a probable damaging effect for both mutations. The p.V599G and the p.W709R mutations are located in the pseudokinase domain of JAK3 essential for its kinase activity as previously reported (Babon, Lucet, Murphy, Nicola, & Varghese, 2014; Chen et al., 2000). To confirm the pathogenicity of these mutations, we performed STAT5 phosphorylation analysis on EBV‐B cells. The results indicated the absence of phospho‐STAT5‐positive population in response to IL2 or IL15 stimulation in patients’ cells compared with HD cells (Figure 4b).

## 4 DISCUSSION

In this study, we described four JAK3‐SCID patients characterized in a period of time of about 10 years using analytical techniques ranging from the basic Sanger sequencing to DHPLC to the most recent NGS. Five novel mutations: the g.13319_13321delTTC and the g.15410_16542del mutations in Pt2, the p.F292V (with the g.15410_16542del) in Pt3, and the p.V599G and the p.W709R in Pt4. *JAK3* gene was analyzed in more than 100 HD and none of the above *novel* mutations were found.

Microsatellites analysis, performed on Pt2‐ and Pt3‐family members to investigate the segregation of the g.15410_16542del mutation, revealed that both patients and their respective carrier parents shared the same haplotype highlighting the hypothesis of a founder mutation. Indeed, Pt2 and Pt3 came from the same small Italian town in the Lazio region of central Italy, so we can suppose that g.15410_16542del mutation might have occurred many generations ago from a single ancestral mutation event and inherited from the same progenitor. Although it would be important to establish the actual frequency of the g.15410_16542del in Central Italy population, we speculate that the presence of two direct Alu repeats, upstream (intron 12) and downstream (intron 13) of the deleted region, induced a mutational mechanism in which the repair of slipped mispairing events during DNA replication caused the deletion (Figure 4). Indeed, it is known that 0.3% of all human inherited disorders, including PID genes, have been associated with germ‐line mutations induced by unequal Alu elements recombination events (Deininger, 2011; Deininger & Batzer, 1999), a percentage that might still be underestimated. Sequence alignment using the Basic Local Alignment Search Tool (BLAST: http://blast.ncbi.nlm.nih.gov) revealed 93% identity between the intron 12 Alu element and the Alu‐Sp and Alu‐Sx subfamily consensus sequences. The Alu element in intron 13 was less conserved (77% with Sx and Sq Alu subfamilies) but long stretches of sequence identity were conserved with the Alu element in intron 12 and this identity probably allowed the unequal recombination (Deininger, 2011; Deininger & Batzer, 1999).

Finally, Pt4 is compound heterozygous for two novel missense substitutions encoding a mutated form of JAK3 protein detectable by WB analysis. This last patient has analyzed by a targeted NGS panel that consented us to make diagnosis. The pathogenicity of these two novel mutations was demonstrated by the absence of phospho‐STAT5‐positive population in response to IL2 or IL15 stimulation in Pt4 compared to HD. In this regard, STAT5 phosphorylation assay is not only useful in suggesting a SCID diagnosis, but it is also a rapid and cost‐effective approach to assess the pathogenicity of new variants (Walshe, Gaspar, Thrasher, Cale, & Gilmour, 2009).

In conclusion, deletions normally detected in PCR‐based analyses in X chromosome might be undetectable in autosomal chromosomes by sequencing technologies including NGS. The new technologies, that is, targeted NGS sequencing, has largely replaced the previous DHPLC and other molecular assays as a better time‐ and cost‐effective approach for routinely diagnostic purposes. On the other hand, other methods, that is, protein expression, functional studies, and molecular analysis of transcripts, are still required and they should be routinely applied to obtain a definitive diagnosis of AR‐SCID in all cases in which only a monoallelic mutation is found particularly for an optimal genetic counseling.

## CONFLICT OF INTEREST

None declared.
