# Supplementary material for: JAK3 mutations in Italian patients affected by SCID: New molecular aspects of a long‐known gene
Source: Mol Genet Genomic Med. 2018 Jul 21;6(5):713–21. doi: 10.1002/mgg3.391 (PMC6160700; doi:10.1002/mgg3.391)
Supplement: Supplementary file 1 [file MGG3-6-713-s001.docx]

**Supplementary Table S1a an S1b.** Gene panels for targeted enrichment and massive sequencing by standard ion PGM 200 Sequencing v2 protocol using Ion 316 v2 chips (Life Technologies).
